# Variation between hospitals in inpatient admission practices for self-harm patients and its impact on repeat presentation

**DOI:** 10.1007/s00127-016-1247-y

**Published:** 2016-06-14

**Authors:** R. Carroll, P. Corcoran, E. Griffin, I. Perry, E. Arensman, D. Gunnell, C. Metcalfe

**Affiliations:** 1 School of Social and Community Medicine, University of Bristol, Canynge Hall, 39 Whatley Road, Bristol, BS8 2PS UK; 2 National Suicide Research Foundation, University College Cork, Cork, Ireland; 3 Department of Epidemiology and Public Health, University College Cork, Cork, Ireland; 4 Department of Obstetrics and Gynaecology, University College Cork, Cork, Ireland

**Keywords:** Self-harm, Medical admission, Psychiatric admission, Instrumental variable, Repetition, Confounding, Suicide

## Abstract

**Purpose:**

Self-harm patient management varies markedly between hospitals, with fourfold differences in the proportion of patients who are admitted to a medical or psychiatric inpatient bed. The current study aimed to investigate whether differences in admission practices are associated with patient outcomes (repeat self-harm) while accounting for differences in patient case mix.

**Methods:**

Data came from the National Self-Harm Registry Ireland. A prospective cohort of 43,595 self-harm patients presenting to hospital between 2007 and 2012 were included. As well as conventional regression analysis, instrumental variable (IV) methods utilising between hospital differences in rates of hospital admission were used in an attempt to gain unbiased estimates of the association of admission with risk of repeat self-harm.

**Results:**

The proportion of self-harm patients admitted to a medical bed varied from 10 to 74 % between hospitals. Conventional regression and IV analysis suggested medical admission was not associated with risk of repeat self-harm. Psychiatric inpatient admission was associated with an increased risk of repeat self-harm in both conventional and IV analyses. This increased risk persisted in analyses stratified by gender and when restricted to self-poisoning patients only.

**Conclusions:**

No strong evidence was found to suggest medical admission reduces the risk of repeat self-harm. Models of health service provision that encourage prompt mental health assessment in the emergency department and avoid unnecessary medical admission of self-harm patients appear warranted. Psychiatric inpatient admission may be associated with a heightened risk of repeat self-harm in some patients, but these findings could be biased by residual confounding and require replication.

## Introduction

Self-harm is a major public health concern and a common reason for people to present to hospital emergency departments [1]. This patient population have a well-established elevated risk of repeat self-harm and suicide [2, 3], yet evidence to support the clinical care of self-harm patients is limited. Randomised control trial (RCT) and observational evidence has accumulated suggesting that psychological therapies, such as cognitive behavioural therapy, may reduce the risk of repeat self-harm and suicide when delivered in outpatient settings [4–7], but there is a lack of robust evidence supporting aspects of care commonly used in the acute management of hospital presenting self-harm.

In the past, admission to a hospital bed was seen as a routine element of self-harm patient management and previous clinical guidelines recommended it for all self-harm patients [8]. While admission is not seen as an intervention in itself, understanding any potential effects it may have on self-harm patient outcomes is important for clinicians and policy makers when making decisions about the configuration of healthcare services. As well as allowing the delivery of clinical care and facilitating psychosocial assessment in settings that do not provide round the clock access to psychiatric services, it has been hypothesised that admission to a hospital bed may have additional therapeutic benefits over and above those associated with treatment. In particular, hospital admission may provide a safe environment which aids recovery away from interpersonal conflict, and service users themselves have reported it as an important aspect of care [9, 10]. Yet despite its potential therapeutic benefits, more recent policy has focused on psychosocial assessment and moved away from an emphasis on hospital admission [11].

Only one RCT has been undertaken assessing the potentially therapeutic impact of hospital admission to a medical bed [9]. This trial randomly allocated low risk self-harm patients who did not clinically require hospitalisation to medical admission or discharge from the emergency room. The study was considerably underpowered and provided no statistical evidence of a beneficial effect of admission (OR 0.75, CI 0.16–3.53). Observational cohort studies have also produced inconsistent results regarding the association of both medical and psychiatric inpatient admission with risk of repeat self-harm and suicide [12, 13]. However, a recent study suggested psychiatric admission in particular may be associated with an increased risk of suicide [14]. These data have led to the suggestion that this association may be causal [15], but the limitations of confounding that are inherent in observational analysis mean these results are difficult to interpret [16].

Conventional observational analyses assessing the effect of admission to a hospital bed, especially psychiatric admission, are likely to be limited due to the effects of confounding by indication. Those patients who are admitted will differ in their characteristics and be at higher risk of poor outcomes, compared to those patients who are not admitted. Instrumental variable analysis offers an alternative approach to overcome problems of confounding by indication when assessing treatment effects in observational data such as these [17].

Naturally occurring variations in patient care, which are well documented in self-harm patients [18, 19], can be used in instrumental variable analyses to estimate the effect of interventions on outcomes and limit bias through confounding. The proportion of self-harm patients who are admitted to a hospital bed has been shown to vary fourfold between hospitals [19]. This variation in hospital admission rates is likely to be related in part to the lack of clear evidence regarding its effectiveness. It is also likely that these variations in management across hospitals are unrelated to the case-mix of presentations but rather reflect local variations in hospital policy, resources and care pathways such as ease of access to liaison psychiatry assessment. These inter-hospital variations in care, that are independent of patient characteristics, have been successfully used in instrumental variable analyses in the past [20]. Such analyses could produce unbiased estimates of the association of hospital admission with risk of repeat self-harm. In the current study we investigated naturally occurring variations in hospital admission of self-harm patients to estimate their association with risk of repeat self-harm using the data from the National Self-Harm Registry Ireland [2, 21].

## Methods

### Data collection

The National Self-Harm Registry Ireland collects information on people presenting to hospital following self-harm [2]. It is one of the only registries worldwide collecting information on hospital presenting self-harm at a national level. For the purposes of data collection, self-harm is defined as “an act with non-fatal outcome in which an individual deliberately initiates a non-habitual behaviour, that without intervention from others will cause self-harm, or deliberately ingests a substance in excess of the prescribed or generally recognised therapeutic dosage, and which is aimed at realising changes that the person desires via the actual or expected physical consequences” [22]. This definition includes people presenting with a range of methods of self-harm including overdose as well as self-injury such as cutting. Data on presentations made by individuals under 16 years of age were excluded. Some institutions (*n* = 5) did not contribute data throughout the full length of follow-up and therefore presentations made at these hospitals were excluded. Furthermore, people who present following accidental overdoses were excluded. We used registry data on index presentations (first presentation in the study period) from 2007 to 2013 for the current analysis (approximately 12,000 attendances per year to 33 hospitals).

### Cohort characteristics

Registry data includes detailed information regarding the demographic and clinical characteristics of self-harm presentations. Information is recorded on age, sex, methods of self-harm, whether alcohol was used as part of the episode and the hospital of attendance. Information is also recorded on the aftercare following hospital presentation. This information included data on whether a patient was admitted to a medical bed, admitted to a psychiatric inpatient bed, or not admitted. Information was unavailable on subsequent aftercare, e.g., whether they received a psychosocial assessment. Furthermore, no information was available on whether patients were transferred to a psychiatric hospital following an initial admission to a medical ward.

### Conventional regression analysis

The main exposures of interest were medical and psychiatric inpatient admission following treatment in the emergency department. The outcome of interest was repeat hospital attendance for self-harm within 12 months of an index presentation. All analyses were based on an individual’s first presentation within 2007–2012 and these patients were followed up until the end of 2013 for any repeat self-harm presentations. Once patients who had a repeat episode within 12 months were identified, their repeat episodes were removed from the dataset.

Two methods were implemented to investigate the association of medical and psychiatric inpatient admission on risk of repeat self-harm: conventional ordinary least squares (OLS) linear regression and instrumental variable two stage least squares regression (IV 2SLS). The main outcome of interest, repeat self-harm, was recorded as a binary variable. OLS linear regression with robust standard errors will produce estimates on the risk difference scale when used with binary outcomes [23]. Using this approach means the OLS analysis was on the same scale as the instrumental variable based estimate.

### Instrumental variable analysis

We used instrumental variable analysis to investigate the association of hospital admission with risk of repeat self-harm independently of the biases associated with confounding by indication. Institutional variation in the proportion of patients admitted to a hospital was investigated as a potential instrument. Variation in a hospital’s preference for a certain treatment has been successfully used in IV analyses in the past [24–26]. Hospitals were categorised into those institutions above or below the median (based on data for all hospitals from 2007 to 2012) institutional admission rate. The instrument was therefore binary with hospitals categorised as either a high or a low admitting hospital.

An effective instrumental variable mimics the process of random allocation in a clinical trial, with the instrumental variable being strongly associated with the exposure (i.e., hospital of attendance should be strongly associated with the likelihood of subsequent hospital admission), but not associated with potential confounders and only associated with the outcome through its effect on the exposure [17]. The instrumental variable analysis will produce a biased effect estimate if the chosen instrument does not meet these criteria. Using hospital as an instrument is based on the assumption that it is random whether someone lives in the catchment area of a hospital that has a high versus low admission rate.

The instrumental variable analysis using hospital as an instrument was undertaken using the Stata user written command “ivreg2” [27]. Potential confounding factors were included in both the OLS and IV model and included age, sex, method of self-harm, use of alcohol in the self-harm episode and hospital level of admission (all variables detailed in Tables 2 and 4). Whether the estimated coefficients from the IV 2SLS analysis differed from the OLS analysis was assessed using the Hausman test [28]. The null hypothesis of the Hausman test is that there are no differences between the regression coefficients of the two models.

### Testing the validity of the IV

One of the key assumptions of an instrumental variable analysis is that the instrument is not associated with potential confounding factors. One means of assessing this is through estimating a prevalence difference ratio (PDR) [29]. The PDR assesses the ability of the instrument to control for confounders and compares that with the level of confounding associated with the exposure. A larger PDR suggests the instrument has been unsuccessful at controlling potential confounding factors. A PDR was calculated for each of the potential confounding factors included in the analysis (age, sex, method of self-harm, use of alcohol and hospital level of admission). In IV analysis the strength of the instrument is related to the amount of variation in the exposure associated with the instrument. In this case the strength of the instrument is the difference in admission rates between hospitals above and below the median admission rate. If the PDR is greater than this difference then the IV analysis is likely to be biased [29]. The association of confounders with the instrument (hospital of attendance) were assessed using both simple descriptive statistics and through the calculation of PDRs.

### Ethics

The National Research Ethics Committee of the Faculty of Public Health, Ireland and the Health Services Ethics Committees of the hospitals included in the registry provided ethical approval for the collection of the data for the study.

## Results

### Cohort characteristics

A total of 43,595 people in Ireland presented to hospital following self-harm during the period studied (2007–2012). The median age was 31 (range 16–94, SD 13.7) and there was a slight predominance of females (52.4 %, 22,840/43,595). Intentional drug overdose was the most common method of self-harm (67.9 %, 29,453/43,595) followed by self-cutting (14.4 %, 6266/43,595). Other high lethality methods including hanging and drowning made up 14.3 % (6231/43,595) of cases, with the remainder using both intentional drug overdose and self-cutting (3.8 %, 1645/43,595).

### Patient care and incidence of repeat self-harm

Overall, 30.6 % (13,326/43,595) of patients were admitted to a medical bed while 9.3 % (4061/43,595) were admitted directly to psychiatric inpatient beds. Altogether 14.8 % (6462/43,595) of the cohort had a repeat episode of hospital presenting self-harm within 12-months of their index presentation regardless of calendar year.

### Medical admission and repeat self-harm

Conventional OLS linear regression suggested there was a small decrease in risk of repetition in those patients who were admitted to a medical bed, compared to those patients who were not medically admitted (RD −0.018, 95 % CI −0.025 to −0.011, Table 1; i.e. 1.8 % fewer patients have a repeat self-harm episode when admitted to a medical bed). However, the observed protective effect was attenuated after controlling for the effects of potential confounding factors (RD 0.000, 95 % CI −0.007 to 0.008; Table 1), in particular method of self-harm and levels of psychiatric admission.

**Table 1:** Ordinary least squares and instrumental variable based estimates of the effect of medical admission on risk of repeat self-harm

	Risk difference^a^ (95 % CI)	*p*	*F* test^b^	Hausman test^c^ (*p*)
Ordinary least squares (OLS)	−0.018 (−0.025 to −0.011)	<0.001	–	–
Adjusted^d^ OLS	0.000 (−0.007 to 0.008)	0.897	–	–
Instrumental variable (IV)	−0.015 (−0.036 to 0.006)	0.153	4518	0.791
Adjusted^d^ IV	−0.009 (−0.030 to 0.012)	0.411	4831	0.353

^a^A positive risk difference (RD) indicates medical admission is associated with increased risk of repeat self-harm, a negative RD indicates a decrease in risk of repeat self-harm

^b^The *F* test gives an indication of the strength of the association between the instrument and the exposure. A *F* value greater than ten can be taken as a crude indication of a potentially strong instrument

^c^The null hypothesis of the Hausman test is that the ordinary least squares RD and the IV RD are the same

^d^Adjusted for age, sex, method of self-harm, use of alcohol and psychiatric admission

The effect of medical admission on risk of repeat self-harm was further investigated with instrumental variable analysis using whether the attendance was made to a high or low admitting hospital as an instrument. The proportion of self-harm patients being medically admitted to a hospital varied from 10 to 74 % across institutions. Hospitals were categorised into those institutions above or below the median (37.9 %) institutional medical admission rate based on data for all hospitals from 2007 to 2012. The proportion of patients being admitted differed by 33.2 % (51.6 vs. 18.4 %) between the two groups of hospitals (those above and below the median institutional admission rate) indicating the instrument was strongly associated to the likelihood of admission.

The unadjusted IV analysis provided some evidence of a protective effect of admission (RD −0.015 95 % CI −0.036 to 0.006) but this estimate was consistent both with a harm and benefit with regard to the effect of admission on risk of repeat self-harm (Table 1). The adjusted IV analysis, as with the OLS analysis, was moderately attenuated when controlling for potential confounders, but the IV analysis still suggested a protective effect of medical admission. Nevertheless, this protective effect of admission was small, consistent with chance, and there was no evidence that the adjusted IV effect estimate differed from that of the conventional regression analysis (Hausman test: *p* = 0.353, Table 1). The attenuation in the adjusted IV effect estimate was related to controlling for the effects of psychiatric admission (effect estimate controlling for psychiatric admission: RD −0.009, 95 % CI −0.030 to 0.013).

The ability of the instrument (whether the attendance was to a high or low admitting hospital) to satisfy the assumption that it is unrelated to potential confounders was investigated by examining the differences in the prevalence of confounders at the two groups of institutions (i.e. hospitals above or below the median institutional admission rate). Reassuringly, the prevalence of patients aged over 35, gender, methods of self-harm and levels of psychiatric admission were similar between the two groups of hospitals (Table 2). This was reinforced by the fact that the PDRs for these factors were all less than the strength of the instrument (33.2 %, the difference in medical admission rates between the two groups of hospitals). However, there was some evidence of imbalances in alcohol consumption between hospitals, with prevalence 4.4 % higher in people attending high admitting hospitals in comparison to those attending low admitting hospital (Table 2).

**Table 2:** Prevalence and prevalence difference ratios associated with medical admission and the instrument of hospital of attendance

	Exposure (*X*)			Instrument (*Z*)			PDR^a^ (%)
	% Not admitted	% Admitted	Prevalence difference (*X*)	% Attended a hospital with a below median admission rate	% Attended a hospital with an above median admission rate	Prevalence difference (*Z*)	
	30,269 (69.4 %)	13,326 (30.6 %)		27,614 (63.3 %)	15,981 (36.7 %)		
Over 35 years %	37.05	47.23	10.18	40.07	40.32	0.25	2.50
Male	48.79	44.93	3.86	47.47	47.86	0.39	−10.10
Method of SH %							
Overdose	61.50	81.32	−19.82	66.93	68.65	1.72	8.70
Self-cutting	18.34	5.37	12.97	14.47	14.20	−0.27	2.10
Other^b^	20.16	13.30	6.86	18.60	17.15	−1.45	21.10
Used alcohol	40.36	43.43	−3.07	39.68	44.10	4.42	144.00
Psychiatric admission	13.42	0.00	13.42	10.41	7.43	−2.98	22.20

^a^ *PDR* Prevalence difference ratio; calculated via [*U*|*Z* = 1] − [*U*|*Z* = 0] / [*U*|*X* = 1] − [*U*|*X* = 0], where *U* risk factor, *Z* instrument, *X* assessed

^b^Other included combined poisoning and self-cutting as well as rare high lethality methods such as hanging and drowning

### Psychiatric admission and repeat self-harm

The same IV used in the investigation of the effects of medical admission was implemented to assess the effect of psychiatric admission on risk of repeat self-harm. Specifically, the IV was whether or not the hospital a patient was attending had an above or below median psychiatric admission rate. The proportion of patients being admitted as psychiatric inpatients varied from 1 to 26 % between hospitals and the median was 9.4 %. The proportion of patients being admitted differed by 9.5 % (14.4 vs. 4.9 %) between the two groups of hospitals (those above and below the median institutional admission rate). This difference is lower (9.4 vs. 33.2 %) than the difference in medical admission rates, suggesting the instrument (whether the hospital is a high or low admitting hospital) was weaker in this analysis.

The highest risk of repeated self-harm was among those receiving psychiatric admission. The rate of repeat self-harm in those patients admitted to a psychiatric bed was 21.6 % compared to 14.1 % in patients not admitted to a psychiatric bed. Conventional regression analysis suggested there was a strong association between psychiatric admission and the risk of repeat self-harm within 12 months of an index presentation (RD 0.075, 95 % CI 0.062–0.088, Table 3). An increased risk was also reported in the IV analysis (RD 0.117, 95 % CI 0.047–0.187; Table 3), but the estimated increased risk associated with psychiatric admission was greater in this analysis. Adjusting for confounders in both the OLS and IV analysis did not attenuate the observed effect estimates.

**Table 3:** Ordinary least squares and instrumental variable based estimates of the effect of psychiatric admission on risk of repeat self-harm

	Risk difference^a^ (95 % CI)	*p*	*F* test^b^	Hausman test^c^ (*p*)
Ordinary least squares (OLS)	0.075 (0.062–0.088)	*p* < 0.001	–	–
Adjusted^d^ OLS	0.073 (0.060–0.087)	*p* < 0.001	–	–
Instrumental variable (IV)	0.117 (0.047–0.187)	0.001	1119	0.231
Adjusted^d^ IV	0.121 (0.046–0.195)	0.001	1181	0.206

^a^A positive risk difference (RD) indicates medical admission is associated with increased risk of repeat self-harm, a negative RD indicates a decrease in risk of repeat self-harm

^b^The *F* test gives an indication of the strength of the association between the instrument and the exposure. A *F* value greater than ten can be taken as a crude indication of a potentially strong instrument

^c^The null hypothesis of the Hausman test is that the ordinary least squares RD and the IV RD are the same

^d^Adjusted for age, sex, method of self-harm, use of alcohol and medical admission

The instrument of whether the hospital had a high or low admission rate again appeared to be unrelated to potential confounders. However, the strength of the instrument in the analysis focused on psychiatric admission was limited (difference in psychiatric admission rates: 9.5 %), therefore, even moderate imbalances in the characteristics of patients between the two groups of hospitals would lead to PDRs greater than the strength of the IV. This was the case for most potential confounders, particularly the use of cutting as a method of self-harm, the prevalence of alcohol use, age and sex (Table 4). The IV estimate may be unreliable due to these imbalances and the small amount of variation in psychiatric admission rates between the two sets of institutions. However, controlling for these potential imbalances did not lead to an attenuation in the IV effect estimate. Furthermore, stratifying the analysis by gender (female only: RD 0.104, 95 % CI −0.006 to 0.215; Male only: RD 0.129, 95 % CI 0.040–0.219), and restricting the analysis by method of self-harm (self-poisoning patients only: RD 0.073, 95 % CI −0.037 to 0.182; Self-injury only: RD 0.166, 95 % CI 0.080–0.253) failed to alter the observed increased risk associated with psychiatric inpatient admission in the IV analyses.

**Table 4:** Prevalence and prevalence difference ratios associated with medical admission and the instrument of hospital of attendance

	Exposure (*X*)			Instrument (*Z*)			PDR^a^ (%)
	% Not admitted	% Admitted	Prevalence difference (*X*)	% Attended a hospital with a below median admission rate	% Attended a hospital with an above median admission rate	Prevalence difference (*Z*)	
	30,269 (69.4 %)	13,326 (30.6 %)		27,614 (63.3 %)	15,981 (36.7 %)		
Over 35 years %	39.37	47.87	8.50	39.65	40.76	1.11	13.06
Male	46.96	53.88	6.92	48.35	46.75	−1.60	−23.12
Method of SH %							
Overdose	69.52	48.49	−21.03	67.28	67.89	0.61	−2.90
Self-cutting	14.29	15.17	0.88	14.66	14.04	−0.62	−70.45
Other^b^	16.19	36.35	20.16	18.06	18.08	0.02	0.10
Used alcohol	42.29	31.69	−10.60	39.39	43.52	4.13	−38.96
Psychiatric admission	33.71	0.00	−33.71	32.86	27.91	−4.95	14.68

^a^ *PDR* Prevalence difference ratio; calculated via [*U*|*Z* = 1] − [*U*|*Z* = 0] / [*U*|*X* = 1] − [*U*|*X* = 0], where *U* risk factor, *Z* instrument, *X* assessed

^b^Other included combined poisoning and self-cutting as well as rare high lethality methods such as hanging and drowning

## Discussion

The impact of admitting a self-harm patient to a hospital bed is not well understood and the frequency of its use varies between hospitals. The proportion of self-harm patients being admitted to a medical bed in the current study was found to vary from 10 to 74 % across hospitals in Ireland. No robust evidence was found to suggest medical admission reduced the incidence of repeat self-harm. This lack of effect of medical admission on risk of repeat self-harm was observed in conventional OLS regression and in IV analysis which was used to overcome problems of confounding by indication. Both the OLS and IV analysis also suggested psychiatric inpatient admission of self-harm patients was associated with an increased risk of repeat self-harm. However, the effects of confounding by indication are of particular concern in the context of psychiatric admission as this is an intervention that is reserved for people within the self-harm patient population who have especially acute mental health needs [30]. The OLS estimate is therefore likely to be biased. The IV estimate may also suffer from residual confounding and further replication is required before claims of a casual association between psychiatric inpatient admission and risk of repeat self-harm are justified.

The lack of association between medical admission and risk of repeat self-harm mirrors null findings from the Multicentre Study of Self-harm in England which found no consistent evidence of an association between medical admission and risk of repeat self-harm [12]. However, these data did suggest that psychosocial assessment of self-harm patients reduced the risk of repeat self-harm and that the potentially protective effect of psychosocial assessment was not mediated by the effects of medical admission. These findings support the results of the current study that suggest, beyond the delivery of appropriate clinical care, medical admission is not an important component of the care pathway for self-harm patients in terms of reducing risk of repeat self-harm.

Not only is there little evidence of a benefit of medical admission on risk of repeat self-harm, but its cost effectiveness also appears limited. For instance, the numbers needed to treat based on the IV effect estimate (ignoring that this estimate is consistent with no difference) suggests 112 extra patients would need to be medically admitted to avoid one repeat attendance. The UK’s National Institute for Health and Care Excellence (NICE) estimate the costs associated with the medical admission of self-harm patients to range from £204 to £4231 [31]. This variation in applicable costs reflects the different treatments required by self-harm patients (e.g., extended intensive care unit admission vs. a short admission to an observational ward before discharge). Patients who are admitted in some hospitals but would not be in others (the patients the current IV effect estimates apply to) are unlikely to have extended length of stay or require ICU admission, therefore, the lower end of the estimated costs of medical admission are likely to apply to our findings. From an economic perspective then, the effectiveness of medical admission appears to be poor given that the estimated costs of avoiding one repeat hospital presenting self-harm episode would be £22,848 (112 × £204) at a minimum. The weak evidence of medical admission’s association with repeat self-harm and its considerable cost provide justification for the policy shift away from an emphasis on medical admission to one focusing on the importance of psychosocial assessment.

Unlike medical admission, psychiatric inpatient admission was strongly associated with an increased risk of repeat self-harm. However, this finding should be interpreted with caution. Psychiatric inpatient admission is reserved for patients who are likely to suffer from greater psychiatric co-morbidities and a higher risk of poor outcomes [16, 32]. Estimating the effect of psychiatric admission independently of these confounding effects is therefore particularly challenging. Information was unavailable on a number of important potential confounders such as presence of a psychiatric diagnosis, whether the patient was currently in contact with mental health services and whether they had a history of previous self-harm. The latter is one of the strongest risk factors for repeat self-harm [33]. A lack of information on these important factors is an important limitation of this study and means that the adjusted OLS estimate is likely to suffer from residual confounding by indication and therefore be unreliable.

The instrumental variable based analysis replicated the association found in the conventional regression, suggesting psychiatric inpatient admission was associated with an increased risk of repeat self-harm. This finding could suggest psychiatric inpatient admission is causally associated with a heightened risk of repeat self-harm. However, it should be emphasised that the IV effect estimate represents a local treatment effect and only applies to those patients whose chances of psychiatric inpatient admission depend on the hospital they attend (i.e., they are affected by the instrument). Therefore, the increased risk of repeat self-harm associated with psychiatric inpatient admission only applies to ‘discretionary’ psychiatric inpatient admissions. This effect estimate does not apply to people who would always be admitted as psychiatric inpatients regardless of the hospital they attend. Even so, the association of psychiatric inpatient admission with risk of repeat self-harm observed in the IV analysis may still be biased by residual confounding.

Nevertheless, if we do accept this finding as suggesting psychiatric admission is causally linked to an increased risk of repeat self-harm, the circumstances and environment associated with psychiatric inpatient admission may underline this potential association. For instance, psychiatric inpatient settings have been described negatively by service users in the past [34]. Furthermore, it has been hypothesised that the stigma and trauma associated with psychiatric inpatient admission, in particular involuntary admission, may increase risk of adverse outcomes such as repeat self-harm and suicide [15]. Alternatively, the observed increased risk may be related to contagion of self-harming behaviour in an inpatient setting. Some research in adolescent patients has suggested psychiatric wards may lead to increases in self-harm behaviour via the process of contagion, even in patients who previously did not engage in suicidal behaviour [35]. However, evidence of this potential pathway has been based on small samples, produced mixed findings [36], and may not be generalised to an adult self-harming population.

### Strengths and limitations

This analysis is strengthened by the National Self-Harm Registry Ireland’s size (*n* > 43,000), prospective design and national coverage. Furthermore, the marked variations in the proportion of self-harm patients being admitted to a hospital bed meant the instrumental variable for medical admission was strong. However, there were a number of important limitations in this study. Firstly, information on whether a patient received a psychosocial assessment during their hospital presentation was unavailable. Previous cohort studies have highlighted mental health assessment as a potentially key intervention in terms of risk of repeat self-harm [37, 38], and the lack of information on this factor limits our findings. Data on a number of other potential confounders including current psychiatric diagnoses were unavailable. This limited our analyses and means we cannot rule out residual confounding by indication, particularly with regard to our analysis of psychiatric inpatient admission. Furthermore, our analysis combined ecological variables (a hospital’s likelihood of admission) with analysis at the patient level. While this can strengthen causal inference, it may also introduce ecological biases that are not present at the individual level [39]. For instance, it has been hypothesised that areas with higher rates of inpatient admission for people with mental illness are likely to have poor provision of community mental health services [40]. If this is the case, the current increased risk of repeat self-harm associated with psychiatric inpatient admission may not reflect its harmful effects but rather that the provision of subsequent community care in the catchment areas of these hospitals is poor. Unfortunately, there were no data available on community mental health service provision available in the Irish registry data to investigate this potential bias.

## Conclusion

The findings of the current study produced no strong evidence to suggest medical admission of self-harm patients reduces the risk of repeat self-harm and supports the shift in the focus of clinical guidelines away from the importance of medical admission to one emphasising psychosocial assessment. Psychiatric inpatient admission of a sub-group of self-harm patients was found to be associated with an increased risk of repeat self-harm using both conventional and instrumental variable based analysis. However, these findings may be biased by the considerable effects of confounding by indication associated with psychiatric inpatient admission and should be interpreted with caution.
